# Finite-Time Line-of-Sight Guidance-Based Path-Following Control for a Wire-Driven Robot Fish

**DOI:** 10.3390/biomimetics9090556

**Published:** 2024-09-15

**Authors:** Yuyang Mo, Weiheng Su, Zicun Hong, Yunquan Li, Yong Zhong

**Affiliations:** Shien-Ming Wu School of Intelligent Engineering, South China University of Technology, Guangzhou 511442, China

**Keywords:** biomimetic robotic fish, path following, line-of-sight guidance law, directional tracking

## Abstract

This paper presents an adaptive line-of-sight (LOS) guidance method, incorporating a finite-time sideslip angle observer to achieve precise planar path tracking of a bionic robotic fish driven by LOS. First, an adaptive LOS guidance method based on real-time cross-track error is presented. To mitigate the adverse effects of the sideslip angle on tracking performance, a finite-time observer (FTO) based on finite-time convergence theory is employed to observe the time-varying sideslip angle and correct the target yaw. Subsequently, classical proportional–integral–derivative (PID) controllers are utilized to achieve yaw tracking, followed by static and dynamic yaw angle experiments for evaluation. Finally, the yaw-tracking-based path-tracking control strategy is applied to the robotic fish, whose motion is generated by an improved central pattern generator (CPG) and equipped with a six-axis inertial measurement unit for real-time swimming direction. Quantitative comparisons in tank experiments validate the effectiveness of the proposed method.

## 1. Introduction

As the demand for marine exploration and development continues to grow, underwater robots have emerged as the preferred choice to replace human intervention to ensure safer and more stable underwater operations. Traditionally, underwater robots have relied primarily on propellers for propulsion. However, propeller-based propulsion systems have several limitations, such as high noise, low efficiency, poor yaw stability [1], and limited maneuverability, which make them unsuitable for certain mission areas. Fish, having evolved over millions of years, demonstrate remarkable underwater locomotion capabilities [2]. Leveraging advances in disciplines such as biology and robotics, researchers have developed bio-inspired robotic fish that mimic the propulsion mechanisms of real fish. Chen et al. introduce a tensioning integral robotic fish and provide a detailed description of its overall structure, stiffness, and mechatronics. The results indicate that the use of tensioning integral nodes can improve swimming performance [3]. A bionic robotic fish driven by ionic polymer–metal composite (IPMC) actuators is presented, and the effect of passive fins on swimming performance is investigated [4]. Michael S et al. [5] were the first to mimic the propulsion mechanism of fish in the presentation of an efficient bionic robotic fish. Du et al. [6] designed a novel tuna-inspired robot with rapid swimming and exceptional maneuverability. Dong et al. designed an innovative bionic gliding robotic fish, which has excellent gliding performance [7]. Liao et al. [8] propose a robotic fish consisting of multiple pulling wires to simulate muscles, as well as an elastic element to simulate the spine of the fish, achieving a swimming speed of 0.54 m/s. Zhong et al. presented a robotic fish featuring an active line-driven body combined with a soft compliant tail for efficient wave swimming, closely resembling real fish behavior, reaching a maximum speed of 2.15 body length/s [9]. These advancements in robotic fish technology demonstrate the potential to overcome limitations associated with traditional underwater propulsion methods.

Early research in the field of robotic fish has primarily focused on the structural design and implementation of locomotion. To further advance the practical applications of bio-inspired robotic fish, current efforts are directed toward enhancing their autonomy and intelligence, with the ultimate goal of improving their operational capabilities and cognitive functions. These endeavors encompass a range of tasks, including water exploration [10], autonomous obstacle avoidance [11,12], kinematic optimization [13], target tracking [14], path planning [15], and path following [16]. These studies are dedicated to developing control strategies that empower robotic fish to effectively handle diverse tasks in underwater environments. In practical applications, robotic fish can be applied to ocean monitoring, undersea operations, aquatic life observation, pollution search, etc. [17], showcasing its immense potential value. Among the various features, path following plays a critical role in enhancing the autonomy and intelligence of bio-inspired robotic fish. It enables them to efficiently perform tasks, such as patrolling and sampling, in underwater environments.

According to existing research, a typical path-following controller consists of two main components, namely a guidance subsystem and an inner-loop controller. The guidance subsystem commonly employs a line-of-sight (LOS) guidance law that aligns the fish’s heading with the line of sight to achieve the desired position. This approach is straightforward and practical and has been widely applied in the control of unmanned surface vessels. Conversely, the inner-loop controller employs various control methods for angle control, such as sliding mode control, PID control, and model predictive control. For example, Liu et al. [18] used a fixed-radius LOS and an improved deep deterministic policy gradients (DDPG) algorithm to control a bio-inspired dolphin to follow a three-dimensional path. Chen et al. [16] employed traditional LOS to achieve real-time desired yaw and pitch angles and used a fuzzy PID controller for tail-joint control and a PID controller for pectoral fin attack-angle control to achieve bio-inspired three-dimensional spiral path tracking. However, the simulation results showed less than ideal tracking performance.

In practical applications, LOS, which relies solely on position error as feedback, may demonstrate reduced tracking performance in the presence of external disturbances. Furthermore, when the lateral tracking error of the robotic fish is zero, regardless of any errors along the path, the desired angle provided by the guidance system will align with the path angle, resulting in deviation from the intended path and consequent degradation of tracking performance. Fossen et al. [19] introduced an integral LOS (ILOS) to compensate for the sideslip angle caused by ocean currents. They utilized an adaptive approach to estimate and compensate for the sideslip angle and demonstrated the effectiveness of the proposed ILOS using the Lyapunov stability theory. In order to overcome the mutual constraint between lateral tracking error and along-track error in traditional LOS, Qu et al. [20] introduced an iterative mechanism to find the optimal path point within a single control cycle to ensure zero along-track error. Through theoretical analysis, an extended-state LOS (ELOS) was derived based on the variable distance to adaptively compensate for the current yaw-tracking error and sideslip angle. Experimental results demonstrated that ELOS surpasses traditional LOS and ILOS in terms of tracking performance. However, its convergence rate is slow, and its observer is complex. Dai et al. [21] proposed a novel path factor update law utilizing a logarithmic-type boundary-layer function (BLF) to limit the along-track error within a constrained range during a single control cycle, effectively addressing the issue of along-track error impeding the convergence of lateral tracking error. These innovative approaches are aimed at enhancing the tracking performance of bio-inspired robotic fish by compensating for the limitations of traditional LOS and mitigating the effects of external disturbances as well as mutual constraints between different tracking errors. However, it should be noted that, in certain cases, automatic compensation for errors was not achieved.

In summary, LOS shows great potential for path-tracking applications. However, traditional LOS methods for path tracking are limited by slow convergence, inadequate tracking accuracy, and significant interference from the sideslip angle of robotic fish. We work on solving the problems of traditional LOS for robotic fish path-tracking applications and the perturbation problems caused by the side-slip angle of robotic fish. Therefore, this study introduces an adaptive LOS (ALOS) control strategy that incorporates a compensation mechanism for the sideslip angle using a finite-time observer (FTO). The results of experiments conducted in a water tank validate the effectiveness of the proposed guidance strategy, showing faster convergence speed and lower steady-state error when compared to traditional guidance methods. The bio-inspired robotic fish achieves precise and stable path tracking, thus confirming the effectiveness of this closed-loop control strategy. 

The paper makes two significant contributions:

(1) A dynamic look-ahead distance adjustment mechanism was adopted to improve the convergence speed and reduce the overshoot of the traditional LOS;
(2) An FTO is proposed to estimate and correct the sideslip angle, effectively solving the path deviation problem.

The remainder of this paper is organized as follows. Section 2 describes the mechanical structure and electrical system of the wire-driven robotic fish. Section 3 presents the kinematic model based on the CPG model, as well as the proposed FTO-based ALOS (FTALOS) as a path-following control guidance subsystem for the robotic fish and stability analysis. Pool experimental validation and result analysis are performed in Section 4. Finally, conclusions and future work are summarized in Section 5.

## 2. Design of Robotic Fish

### 2.1. Mechanical Design

Fish in their natural environment exhibit a remarkable level of body flexibility. During locomotion, the fish’s body adopts a sinusoidal pattern, forming an “S” shape, which has been scientifically proven to optimize the transmission of swimming waves and enhance movement stability [2]. Biologists have utilized electromyography to confirm that the majority of the fish’s swimming energy is produced by the muscles in the anterior and middle sections of the body, with the muscles in the caudal fin serving as transmitters. In other words, the anterior and middle sections act as an engine for oscillatory motion, propelling the posterior half like a propeller [22,23]. During tail swinging, the fish exhibits lateral deviation of its posterior body to one side, while the tail and caudal fin bend towards the opposite side as a result of fluid resistance in the water, leading to a distinctive “S” shape. This observation implies that fish do not exert intentional control over their caudal fin to counter water resistance. Instead, the fin flexes autonomously in response to inertia, contributing to increased efficiency.

**Figure 1:**
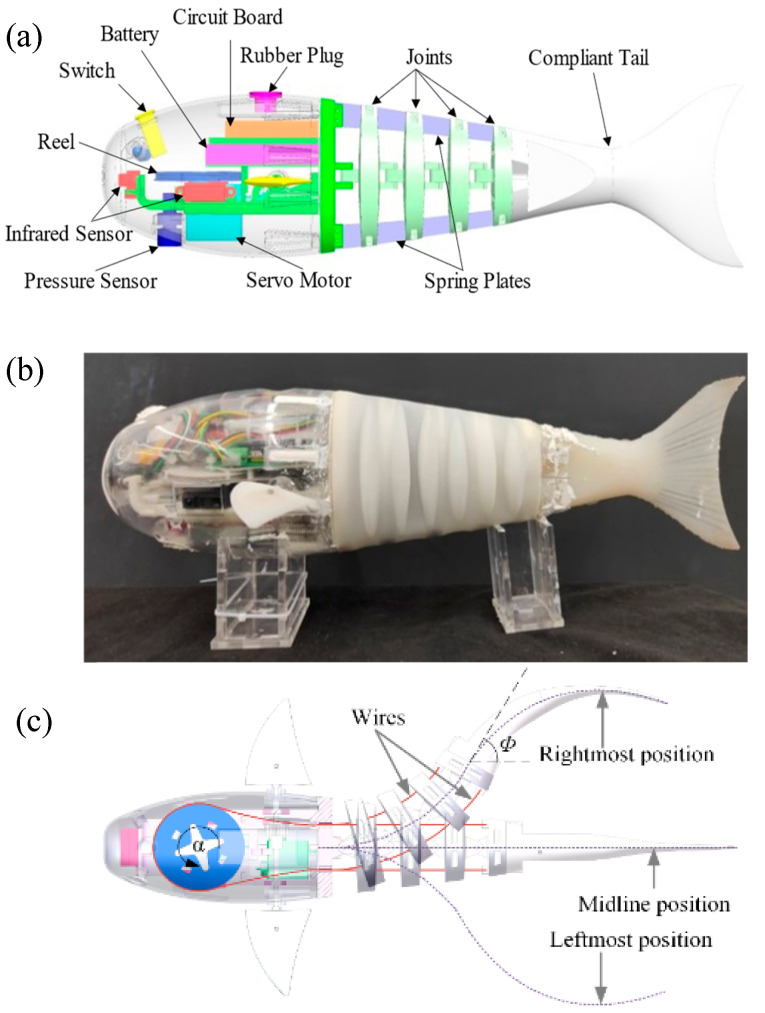
Developed wire-driven active body and compliant tail biomimetic robotic fish. (**a**) Overall CAD structure; (**b**) prototype; and (**c**) illustration of the swinging motion of the robotic fish.

Drawing inspiration from the physiological structure and propulsion mechanism of real fish, we have proposed a segmented robotic fish design (Figure 1a,b) consisting of a rigid head, a movable driving component, and a flexible tail fin. The active driving component includes a primary servo motor located in the head, along with a movable body section. In contrast, as shown in Figure 1c, the flexible component constitutes a passive flexible tail fin. The amalgamation of two components gives rise to an active compliant propulsion mechanism that closely mimics the physiological structure of actual fish.

### 2.2. Electronics

The electronic components used in the robotic fish are shown in Figure 1a and Table 1. We utilize the STM32F103 as the microcontroller for the robotic fish, and the E62-433T20D as the communication module, which operates at a frequency of 433 MHz with a data transmission baud rate of 115,200 bits/s, facilitating effective underwater communication. Additionally, the power source driving the caudal fin’s movement is the SAVOX SW-1210SG servo, which is capable of providing an ultra-high torque of 3.2 N∙m and oscillating at a frequency of 2 Hz under load, meeting the requirements for the normal swimming behavior of the robotic fish. The robotic fish is also equipped with a six-axis sensor MPU6050, primarily used to measure the real-time yaw angle of the fish. All electronics are powered by a 7.4 V DC lithium battery. 

## 3. CPG-Based Path Following Control Design

### 3.1. CPG-Base Model

#### 3.1.1. CPG Model

The rhythmic tail-swinging motion observed in natural fish is driven by a CPG and dynamically modulated by sensory inputs, such as water flow, temperature, pressure, and olfactory and visual information. This movement aids fish in adapting to changes in water flow and aquatic environments. Based on meticulous observations of actual fish movement, we propose an enhanced CPG model that is suitable for the proposed wire-driven biomimetic robotic fish. The model is derived from Ijspeert’s successful CPG model applied to gait generation control in biomimetic salamanders [24,25]. Manduca et al. [26] used a CPG model to control the torque on the caudal fin. Korkmaz et al. [27] employed the CPG model, fuzzy logic, and a sensory feedback mechanism to achieve three-dimensional motion. Given that our robotic fish has only one motor drive, we simplify the CPG model by removing complex coupling and introducing the concept of temporal ratio to describe varying durations in recovery and flapping phases during turning. The final expression of the CPG model is as follows:

(1) $$\left\{\begin{array}{l} \ddot{b}=k_{b}\left(0.25k_{b}\left(B-b\right)-\dot{b}\right)\\ \ddot{m}=k_{m}\left(0.25k_{m}\left(M-m\right)-\dot{m}\right)\\ \dot{\phi }=[\frac{{\left(1+R\right)}^{2}}{4R}-\frac{R^{2}-1}{4R}sign(sin(\phi )]\omega \\ \alpha =b+mcos\left(\phi \right)\;\\ \beta =b+msin\left(\phi \right)\\ sign\left(\lambda \right)=\left\{\begin{array}{c} \;\;\;1,\;\;\;if\;\lambda >0\\ \;\;\;\;0,\;\;\;if\;\lambda =0\\ -1,\;\;\;if\;\lambda <0 \end{array}\right. \end{array}\right.$$

where, b, m, and ϕ represent the real-time values of the caudal fin beating bias, amplitude, and phase, while (M,B,ω,R) represent the beating amplitude, bias, frequency, and time ratio taken for one complete cycle in the CPG high-level control commands. Additionally, $k_{b}$ and $k_{m}$ are design parameters used to control the sensitivity of the CPG commands. The time ratio is defined as the ratio between the restore phase ($t_{r}$) and the beat phase ($t_{b}$). This parameter can be utilized to control the turning rates. By adjusting the parameters in the high-level control commands of CPG, different swimming modes can be generated, and smooth transitions between different modes can be achieved without sudden changes in the control variables.

The first and second differential equations represent second-order linear differential equations, indicating that the current amplitude m converges to the CPG input M. The rate of convergence is contingent upon the coefficient $k_{m}$, while the offset b exhibits similar behavior. When given inputs M>0 and ω>0, an oscillatory gait is initiated, at which point a non-zero input b begins to polarize.

#### 3.1.2. Kinematic Model

**Figure 2:**
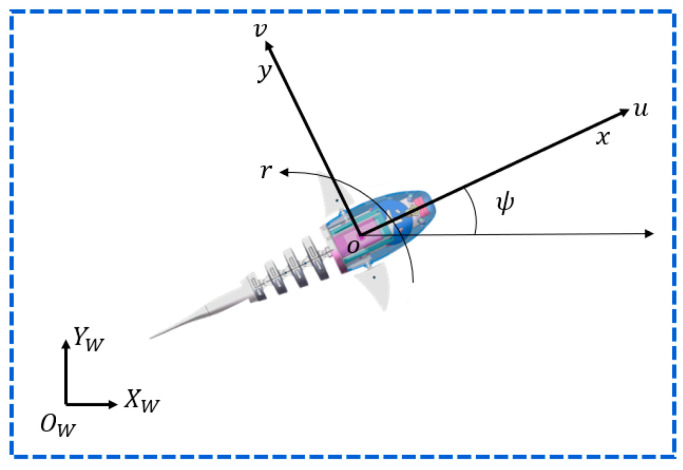
Kinematic diagram of robotic fish.

The kinematic model diagram is shown in Figure 2. Due to the fact that robotic fish typically do not engage in long-term depth adjustments, and adjustments to weight distribution are made during design to ensure stability in the roll direction during swimming, depth variations can be disregarded, and the roll angle and pitch angle can be set to zero, resulting in the simplified two-dimensional planar kinematic equation as follows:

(2) $$\left\{\begin{array}{l} {\dot{X}}_{f}=ucos\psi -vsin\psi \\ {\dot{Y}}_{f}=usin\psi +vcos\psi \\ \dot{\psi }=r \end{array}\right.$$

where $X_{W}O_{W}Y_{W}$ stands for the world coordinate system which is stationary, and $X_{f}$ and $Y_{f}$ represent the position of the robotic fish in the world coordinate system. xoy denotes the coordinate system bound to the motion of the robotic fish, whose direction is shown in Figure 2, and the velocity of the robotic fish in the x-axis and y-axis are u and v, respectively. Meanwhile, r denotes the angular velocity of rotation around the z-axis. ψ represents the yaw angle of the robotic fish, i.e., the angle between the x-axis of the robotic fish’s motion coordinate system and the $X_{W}$-axis of the world coordinate system.

#### 3.1.3. Combined Motion

**Figure 3:**
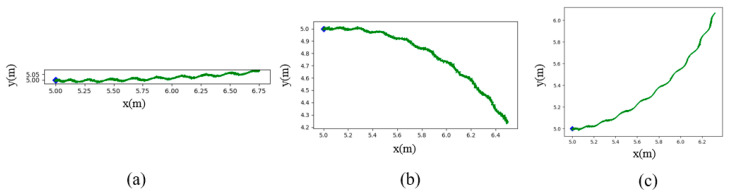
Motion curves of robotic fish under different CPG inputs. (**a**) Straight swimming: M=1.07, w=6.28, b=0, R=1; (**b**) turn right: M=1.07, w=6.28, b=0.1, R=1; and (**c**) turn left: M=1.07, w=6.28, b=−0.1, R=1.

This subsection describes how the CPG model drives the robotic fish to perform motion. The CPG model, as presented in Equation 1, is capable of generating stable swimming vibration gaits of the caudal fin. Theoretically, the swinging caudal fin will generate the force to be applied to the dynamics model, and the generalized acceleration generated by the dynamics model will be integrated to get the generalized velocity, which will be applied to the kinematic model, thus driving the robotic fish to move. However, due to the complex structure of the highly propulsive robotic fish designed in this paper and the complexity of fluid mechanics, it is difficult to establish an accurate dynamics model. So, this paper does not establish a dynamics model but assumes that the acceleration process of the robotic fish is very short and that the robotic fish can soon reach equilibrium. That is, the total propulsive force is equal to the drag force. Therefore, the dynamics model can be ignored, and the mapping relationship from the CPG input to the motion state of the robot fish, i.e., the kinematic model, can be established. When the CPG input parameters are M,w>0, b=0,and R=1, the caudal fin of the robotic fish oscillates symmetrically along the central axis, which produces forward thrust, i.e., u>0 in Equation 2, and the average angular velocity r is zero. Therefore, the robotic fish swims forward, as shown in Figure 3a. While the input is b ≠ 0, the caudal fin of the robotic fish is polarized, thus generating a lateral force, and the angular velocity r ≠ 0, as shown in (b) and (c) of Figure 3. It can be seen that the purpose of controlling the motion of the robotic fish is thereby achieved by changing the input of the CPG.

### 3.2. Outer-Loop Controller Design

**Figure 4:**
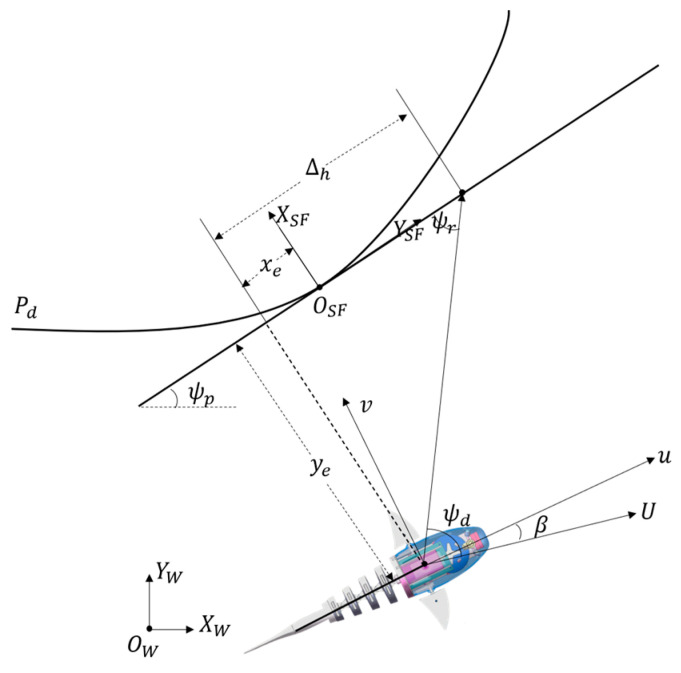
Line-of-sight guidance principle for curved path.

To facilitate description, let us establish a coordinate system as shown in Figure 4. The desired path is defined as $P_{d}$ = ($x_{d}$(ϖ), $y_{d}$(ϖ)), where ϖ is a coefficient related to the path. Taking a point on the desired path as the origin and its tangential direction as the X-axis, we establish the $frame_{SF}$ coordinate system. Based on the geometric relationships in Figure 4, the tracking error of the robotic fish can be expressed in the $frame_{SF}$ coordinate system as follows:

(3) $$e=\left[\begin{array}{c} x_{e}\\ y_{e} \end{array}\right]=R^{T}\left(\psi_{P}\right)\left(P-P_{d}\right)$$

where $R(\psi_{P})$ represents the rotation matrix between the world coordinate system and the $frame_{SF}$ coordinate system, and it is defined as:

(4) $$R\left(\psi_{P}\right)=\left[\begin{array}{cc} \cos\left(\psi_{P}\right) & -\sin\left(\psi_{P}\right)\\ \sin\left(\psi_{P}\right) & \cos\left(\psi_{P}\right) \end{array}\right]$$

where $\psi_{P}$ is the tangent angle or path angle at the current path point, which can be defined as follows. Assuming that $x_{d}(\varpi )$ and $y_{d}(\varpi )$ are twice differentiable with respect to ϖ

(5) $$\psi_{P}\left(\varpi \right)=atan2\left({\dot{y}}_{d}\left(\varpi \right),\;{\dot{x}}_{d}\left(\varpi \right)\right)$$

To achieve path following for the robotic fish, a certain guidance method is required to steer the fish towards the desired path and ensure it swims along that path. In the traditional LOS, the desired heading angle is defined as:

(6) $$\begin{array}{ll} \psi_{d} & =\psi_{P}+\psi_{r}\\ & =\psi_{P}+\mathrm{atan}\left(-\frac{y_{e}}{\Delta_{h}}\right) \end{array}$$

By causing the controlled object to move according to the desired heading angle mentioned above, the controlled object can eventually converge to the desired path. However, the traditional line-of-sight guidance law has three drawbacks:

(1) coupling and mutual constraint exist between the convergence of along-track and cross-track errors;
(2) when the cross-track error is zero, the desired heading angle is outputted by the guidance subsystem equals the path angle;
(3) the constant and relatively large lookahead distance results in a slow convergence speed.

Due to drawback 1, the along-track tracking error of the robotic fish may not be zero throughout the entire motion process, and it can be comparable to the cross-track error [20]. When the desired path is a straight line, only a zero cross-track error is required for the robotic fish to follow the intended path. However, when the desired path is a curved path, both components in two directions must be zero simultaneously for successful path following. Therefore, the fish may have difficulty in accurately implementing path following. In fact, T. Fossen et al. have shown that, for a non-closed path, there exists an optimal path point $P(\varpi^{*})$ where the along-track tracking error $x_{e}$ is zero [19]. For a closed path, multiple optimal path points may exist at the same time, and in this case, the one closest to the previous optimal path point is selected. Therefore, the optimal path point within a single control cycle can be obtained using the following equation:

(7) $$\begin{array}{c} \varpi^{*}=\arg\underset{\varpi \geq 0}{\min}\left(\dot{\varpi }\right)\\ s.t.\;\;\;y-y_{d}\left(\varpi \right)=-\frac{1}{\tan\left(\psi_{P}\left(\varpi \right)\right)}\left(x-x_{d}\left(\varpi \right)\right) \end{array}$$

For drawback 2, when the cross-track error reaches zero, regardless of the magnitude of the along-track error, the desired yaw-angle output from the guidance subsystem will be strictly equal to the path angle. In this case, if the along-track error is non-zero, the angle will guide the robotic fish toward the desired path. However, when the along-track error is zero, indicating that the robotic fish is already on the desired path, it will cause the robotic fish to deviate from the desired path, thus affecting the path-following performance. In the traditional LOS approaches, the derivation process commonly disregards the influence of the sideslip angle. Despite its typically small magnitude, it becomes significant when the cross-track tracking error of the robotic fish nears zero and cannot be overlooked. Moreover, the sideslip angle can provide a certain heading angle compensation to mitigate the deviation phenomenon in the traditional law. Therefore, we proposed a side-slip angle observer to correct the output of the guidance system and improve the path-following performance of the robotic fish.

By differentiating Equation (3), the tracking-error kinematics can be obtained as follows:

(8) $$\left\{\begin{array}{c} {\dot{x}}_{e}=-U_{P}+Ucos\left(\psi -\psi_{P}\left(\varpi \right)+\beta \right)+y_{e}{\dot{\psi }}_{P}\\ {\dot{y}}_{e}=Usin\left(\psi -\psi_{P}\left(\varpi \right)+\beta \right)-x_{e}{\dot{\psi }}_{P}\;\;\;\;\;\;\;\;\;\;\;\;\;\; \end{array}\right.$$

where $U_{P}$ represents the velocity of the path point, U is the real-time synthesized motion velocity of the robotic fish; and β is the sideslip angle which is defined as the angle between U and u, i.e., atan(v/u). By calculating the optimal path point $P(\varpi^{*})$ that minimizes $x_{e}$ according to Equation (7) and assuming that sinβ≈β and cosβ≈ 1, based on the fact that beta is generally <10°. At the same time, it is assumed that the side-slip angle of the robotic fish changes slowly during the movement, i.e., there exists $\dot{\beta }$ = 0. The equation can be reduced to:

(9) $$\begin{array}{ll} {\dot{y}}_{e} & =Usin\left(\psi -\psi_{P}+\beta \right)\\ & =Usin\left(\psi -\psi_{P}\right)cos\beta +Ucos\left(\psi -\psi_{P}\right)sin\beta \\ & \approx Usin\left(\psi -\psi_{P}\right)+Ucos\left(\psi -\psi_{P}\right)\beta \end{array}$$

The side-slip angle observer can be designed as below:

(10) $$\left\{\begin{array}{l} {\dot{\hat{y}}}_{e}=Usin\left(\psi -\psi_{P}\right)+Ucos\left(\psi -\psi_{P}\right)\;\hat{\beta }-\lambda sig^{0.5}\left({\tilde{y}}_{e}\right)\\ \dot{\hat{\beta }}=-Ucos\left(\psi -\psi_{P}\right){\tilde{y}}_{e}\;\;\;\;\;\;\;\;\;\;\;\;\;\;\;\;\; \end{array}\right.$$

where ${\hat{y}}_{e}$ is the estimated value of $y_{e}$, and ${\tilde{y}}_{e}={\hat{y}}_{e}–y_{e}$ represents the estimation error of $y_{e}$. $\hat{\beta }$ is the estimated value of the side-slip angle β, and $sig^{0.5}\left(x\right)={\left|x\right|}^{0.5}sign\left(x\right)$ denotes the square root sign function. With the action of this observer, the estimation errors ${\tilde{y}}_{e}$ and $\tilde{\beta }$ will converge to zero, i.e., ${\hat{y}}_{e}\to y_{e}$ and $\hat{\beta }\to \beta$.

**Proof.**  Subtract Equation (9) from Equation (10), and we can get:

(11) $${\dot{\tilde{y}}}_{e}=Ucos\left(\psi -\psi_{P}\right)\tilde{\beta }-\lambda sig^{0.5}\left({\tilde{y}}_{e}\right)$$

Choosing the Lyapunov function as $V_{1}=\frac{1}{2}({\tilde{y}}_{e}^{2}+{\tilde{\beta }}^{2})$ and taking the derivative of $V_{1}$:

(12) $$\begin{array}{ll} {\dot{V}}_{1} & ={\tilde{y}}_{e}{\dot{\tilde{y}}}_{e}+\tilde{\beta }\dot{\hat{\beta }}\\ & ={\tilde{y}}_{e}\left(Ucos\left(\psi -\psi_{P}\right)\tilde{\beta }-\lambda sig^{0.5}\left({\tilde{y}}_{e}\right)\right)-Ucos\left(\psi -\psi_{P}\right){\tilde{y}}_{e}\tilde{\beta }\\ & =-\lambda {\tilde{y}}_{e}sig^{0.5}\left({\tilde{y}}_{e}\right)\\ & =-\lambda {\left|{\tilde{y}}_{e}\right|}^{1.5}\\ & =-\lambda {\left|{\tilde{y}}_{e}\right|}^{1.5}-{\lambda \left|\tilde{\beta }\right|}^{1.5}+{\lambda \left|\tilde{\beta }\right|}^{1.5}\; \end{array}$$

With **Lemma 1** in [28], let $\left(a_{1},a_{2},a_{3},\cdots \;,\;a_{n}\right)$ $\in R^{n}$ and 0<ρ<2. Then, the following inequality holds:

(13) $${\left(a_{1}^{2}+a_{2}^{2}+a_{3}^{2}\cdots +a_{n}^{2}\right)}^{\rho }\leq {\left(a_{1}^{\rho }+a_{2}^{\rho }+a_{3}^{\rho }+\cdots +a_{n}^{\rho }\right)}^{2}$$

So that:

(14) $$\begin{array}{ll} {\dot{V}}_{1} & =-\lambda \left({\left|{\tilde{y}}_{e}\right|}^{1.5}+{\left|\tilde{\beta }\right|}^{1.5}\right)+\lambda {\left|\tilde{\beta }\right|}^{1.5}\\ & \leq -\lambda {\left({\left|{\tilde{y}}_{e}\right|}^{2}+{\left|\tilde{\beta }\right|}^{2}\right)}^{\frac{1.5}{2}}+\lambda {\left|\tilde{\beta }\right|}^{1.5}\\ & =-2^{\frac{1.5}{2}}\lambda V_{1}^{\frac{1.5}{2}}+\lambda {\left|\tilde{\beta }\right|}^{1.5} \end{array}$$

It is noted that there should exist a constant $\theta_{0}$ satisfying $0<\theta_{0}<1$, such that $2^{\frac{1.5}{2}}\lambda \theta_{0}V^{\frac{1.5}{2}}=\lambda \theta_{0}{\left({\tilde{y}}_{e}^{2}+{\tilde{\beta }}^{2}\right)}^{\frac{1.5}{2}}=\lambda {\left|\tilde{\beta }\right|}^{1.5}$, which implies $\lambda {\left|\tilde{\beta }\right|}^{1.5}-2^{\frac{1.5}{2}}\lambda \theta_{0}V^{\frac{1.5}{2}}=0$. Therefore, we can derive the following equation:

(15) $$\begin{array}{ll} {\dot{V}}_{1} & \leq \gamma_{\beta }-2^{\frac{1.5}{2}}\lambda \theta_{0}V_{1}^{\frac{1.5}{2}}-2^{\frac{1.5}{2}}\lambda \left(1-\theta_{0}\right)V_{1}^{\frac{1.5}{2}}\\ & =-2^{\frac{1.5}{2}}\lambda \left(1-\theta_{0}\right)V_{1}^{\frac{1.5}{2}} \end{array}$$

According to **Lemma 2** in [28], for the nonlinear autonomous system $\dot{x}=f(x)$, $f\left(0\right)=0$, if there exists a positive definite continuously differentiable scalar function $V\left(x\right)$, $R^{n}\to R$ satisfies the following conditions:

(16) $$\dot{V}\left(x\right)\leq -\alpha V^{\rho }\left(x\right)$$

where α>0, 0<ρ<1. Then, the system is finite time stable at the origin, i.e., there exists a time *T*:

(17) $$T\leq \frac{1}{\alpha \left(1-\rho \right)}V^{1-\rho }\left(x\right)$$

such that $V\left(x\left(t\right)\right)=0,\;\forall t>T$.

The estimation error $\left({\tilde{y}}_{e},\tilde{\beta }\right)$ will converge to (0,0) within a finite time, and the convergence time $T_{1}$ satisfies the following condition:

$$T_{1}\leq \frac{V_{1}{\left(0\right)}^{\frac{1.5}{2}}}{2^{\frac{1.5}{2}}\lambda \left(1-\theta_{0}\right)\left(1-\frac{1.5}{2}\right)}$$

Therefore, under the action of the observer, we can approximate ${\hat{y}}_{e}$ to $y_{e}$ and $\hat{\beta }$ to β. Based on the estimated value of the sideslip angle obtained from the observer, the yaw control law of the guidance subsystem is given by:

(18) $$\begin{array}{ll} \psi_{d} & =\psi_{r}+\psi_{p}\\ & =\mathrm{atan}\left(-\frac{y_{e}}{\Delta_{h}}-\hat{\beta }\right)+\psi_{p} \end{array}$$

Under the influence of this desired yaw angle, the robotic fish’s lateral error can converge to zero. □

**Proof.**  Assuming that the robotic fish can perfectly track the desired yaw angle, when the desired yaw angle is substituted into the observer, the following can be obtained:

(19) $$\begin{array}{ll} {\dot{y}}_{e} & =-\frac{U\left(y_{e}+\hat{\beta }\Delta_{h}\right)}{\sqrt{\Delta_{h}^{2}+{\left(y_{e}+\hat{\beta }\Delta_{h}\right)}^{2}}}+\frac{U\beta \Delta_{h}}{\sqrt{\Delta_{h}^{2}+{\left(y_{e}+{\hat{\beta }\Delta }_{h}\right)}^{2}}}\\ & =-\frac{Uy_{e}}{\sqrt{\Delta_{h}^{2}+{\left(y_{e}+\hat{\beta }\Delta_{h}\right)}^{2}}} \end{array}$$

Taking the Lyapunov function as $V_{2}=\frac{1}{2}y_{e}^{2}$ and differentiating it, it can be derived that:

(20) $$\begin{array}{ll} {\dot{V}}_{2} & =y_{e}{\dot{y}}_{e}\\ & =-\frac{Uy_{e}^{2}}{\sqrt{\Delta_{h}^{2}+{\left(y_{e}+\Delta_{h}\hat{\beta }\right)}^{2}}}\\ & \leq -kV_{2}\\ & \leq 0\; \end{array}$$

where $k=U/\sqrt{\Delta_{h}^{2}+{\left(y_{e}+\Delta_{h}\hat{\beta }\right)}^{2}}$. According to the Lyapunov stability theory, it can be concluded that $y_{e}$ will eventually converge to zero.

**Figure 5:**
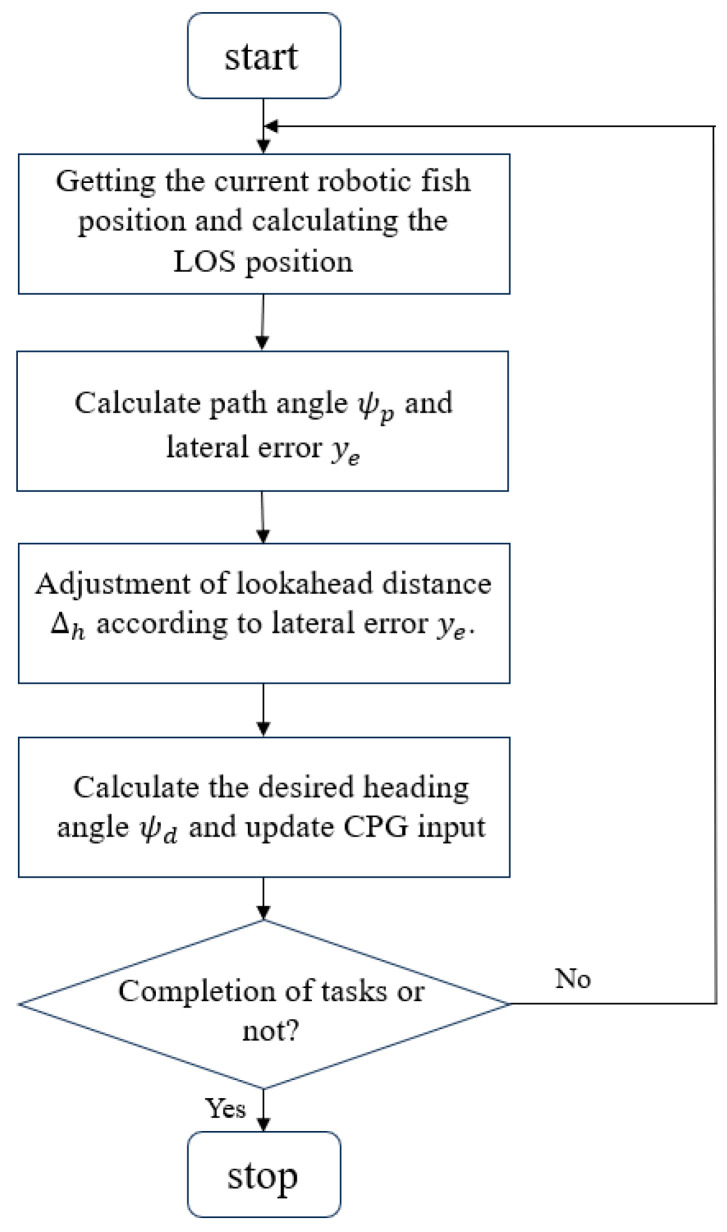
Flowchart of the working of ALOS.

To address drawback 3, the selection of the lookahead distance plays a crucial role. Thus, an adaptive LOS strategy is proposed. When encountering a large lateral tracking error $y_{e}$, indicating that the fish is significantly deviating from the desired path, it is advantageous for the fish to swiftly approach the desired path. In this case, opting for a smaller lookahead distance would result in the desired yaw angle being nearly perpendicular to the path angle, thereby enabling faster convergence with the desired path. On the other hand, conversely, when facing a small lateral tracking error, it is preferable to utilize a larger lookahead distance in order to minimize overshooting during convergence. In this scenario, emphasis should be placed on aligning the desired yaw angle with the path angle. It is important to note that, while a smaller lookahead distance facilitates convergence speedily, an excessive focus on convergence can lead to oscillations. Therefore, employing a time-varying formula for calculating lookahead distance can mitigate these issues:

(21) $$\Delta_{h}=\left(\Delta_{h_{\max}}-{\Delta_{h}}_{\min}\right)\left(-\tanh^{2}\left(ky_{e}\right)+1\right)+{\Delta_{h}}_{min}$$

where $\Delta_{hmax}$ represents the upper limit of the time-varying lookahead distance, while $\Delta_{h\min}$ represents the lower limit. The parameter k serves as a positive design parameter that controls the sensitivity of the lookahead distance to the lateral tracking error. By modifying the value of k, the sensitivity of the lookahead distance to variations in the lateral tracking error can be regulated, which facilitates the trade-off of convergence speed and oscillations during the tracking process. The flowchart of ALOS is shown in Figure 5.

**Figure 6:**
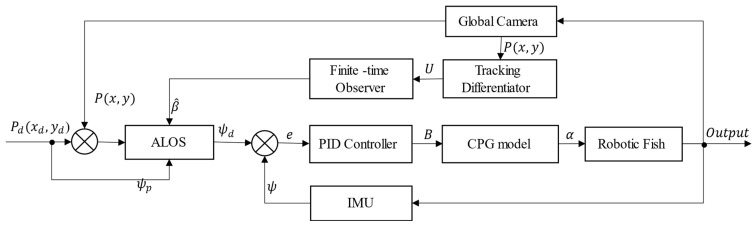
Control diagram of path-following control for the wire-driven robotic fish. □

Figure 6 illustrates the overall framework of the FTALOS control method for path tracking.

(22) $$\left\{\begin{array}{l} fh=fhan\left(x_{1}\left(k\right)-v\left(k\right),x_{2}\left(k\right),r,h\right)\\ x_{1}\left(k+1\right)=x_{1}\left(k\right)+hx_{2}\left(k\right)\\ x_{2}\left(k+1\right)=x_{2}\left(k\right)+hfhan \end{array}\right.$$

where v(k) is the real-time position of the robotic fish, $x_{1}(k)$ is the tracking signal of v(k), $x_{2}(k)$ is the tracking signal of $\dot{v}(k)$, and fhan is the optimal control synthesis function, which is defined as [28]:

(23) $$\left\{\begin{array}{l} d=rh\\ d_{0}=dh\\ a_{0}=hx_{2}\\ y=x_{1}+a_{0}\\ a_{1}=\sqrt{d\left(d+8\left|y\right|\right)}\\ a_{2}=a_{0}+sign\left(y\right)\left(a_{1}-d\right)/2\\ a=\left(a_{0}+y\right)fsg\left(y,d\right)+a_{2}\left(1-fsg\left(y,d\right)\right)\\ fhan=-r\left(\frac{a}{d}\right)fsg\left(a,d\right)-rsign\left(a\right)\left(1-fsg\left(a,d\right)\right)\\ fsg\left(x,d\right)=\left(sign\left(x+d\right)-sign\left(x-d\right)\right)/2 \end{array}\right.$$

### 3.3. Inner-Loop Controller Design

In the previous section, the primary function of the outer-loop controller is to simplify the path-tracking control problem by transforming it into a lateral tracking-error stabilization control problem. This is further enhanced through the implementation of an improved LOS, which subsequently converts it into a yaw-tracking control problem. Therefore, an effective yaw-tracking controller is necessary, involving the utilization of LOS as the outer-loop controller for deriving the desired yaw-angle signal. Subsequently, the inner-loop yaw-tracking controller generates the corresponding motor control signal, thereby driving the robotic fish to accurately follow the intended path.

The compliant and flexible design of the tail in the proposed biomimetic robotic fish structure in Section 2 enhances energy efficiency while achieving natural fish-like swimming. However, accurately establishing the dynamic model of the robotic fish presents challenges, making the application of model-based control methods impractical. Conversely, PID controllers have been widely employed in various control tasks due to their simplicity, robustness, and ability to achieve satisfactory control performance without requiring a precise model of the controlled system. Therefore, we utilize a PID controller as the inner-loop yaw-tracking controller, with its specific control output defined as follows:

(24) $$\begin{array}{ll} B\left(k\right) & =K_{p}e\left(k\right)+K_{i}T_{s}\sum_{i=1}^{k}e\left(i\right)+K_{d}\frac{e\left(k\right)-e\left(k-1\right)}{T_{s}}\\ & =K_{P}e\left(k\right)+K_{I}\sum_{i=1}^{k}e\left(i\right)+K_{D}\left(e\left(k\right)-e\left(k-1\right)\right) \end{array}$$

where B is bias, one of the inputs of the CPG model. $e\left(i\right)=\psi \left(i\right)-\psi_{d}(i)$ represents the tracking error at the i-th time instant, where ψ(i) is the current yaw angle and $\psi_{d}(i)$ is the desired yaw angle. The parameters $K_{p}$, $K_{i}$, and $K_{d}$ are the proportional, integral, and derivative gains of the controller, respectively. $T_{s}$ represents the sampling period.

To improve the robustness of the controller, an incremental PID approach is employed. By taking the difference of Equation (24), we obtain:

(25) $$\begin{array}{ll} \Delta B= & K_{P}\left(e\left(k\right)-e\left(k-1\right)\right)+K_{I}e\left(k\right)\\ & +K_{D}\left(e\left(k\right)-2e\left(k-1\right)+e\left(k-2\right)\right) \end{array}$$

The inclusion of the integral term introduces the potential for integral windup. As a result, it is essential to evaluate whether the control signal has reached saturation when calculating the adjustment to the control signal. If saturation is identified, the integral component is disregarded, and only the proportional and derivative terms are retained. This approach, commonly known as an anti-windup mechanism, effectively mitigates the negative impacts of integral saturation.

It is important to note that the MPU6050 obtains the current orientation angles through velocity integration, where the Z-axis angle represents a relative angle rather than an absolute angle. Upon power-up, the Z-axis angle is initialized to 0°, indicating that the fish’s head direction at power-on is considered the 0° direction. Typically, the range of variation for the Z-axis angle is [−180°, 180°]. Therefore, significant angle jumps may occur when the fish’s yaw direction is opposite to the initial direction at power-up, such as a jump from 180° to −180° or from −180° to 180°. In order to mitigate abrupt changes in the yaw-tracking error and prevent erroneous control commands due to angle discontinuities, it is essential to preprocess the yaw angle obtained from the fish’s attitude sensor before updating the control signal. The specific preprocessing steps are as follows:

(26) $$e=\left\{\begin{array}{cc} e-2\pi & ,\;\mathrm{if}\;e\geq \pi \\ e\;\;\;\;\;\; & ,\;\mathrm{if}-\pi <e<\pi \\ e+2\pi & ,\;\mathrm{if}\;e\leq -\pi \end{array}\right.$$

Note that the Z-axis angle sampled by the MPU6050 is not the actual yaw angle but the direction in which the fish head is pointing because the fish head will oscillate during the swimming process. In order to get the actual yaw angle, we need to approximate the yaw angle by first-order filtering:

(27) $$\psi_{act}\left(k\right)=\alpha \psi_{act}\left(k-1\right)+\left(1-\alpha \right)\psi_{IMU}\left(k\right)$$

where α is the filtering factor,$\;\psi_{act}(k)$ is the yaw angle corresponding to the current sampling cycle, $\psi_{act}(k-1)$ is the yaw angle of the previous cycle, and $\psi_{IMU}(k)$ is the pointing of the head of the fish captured by the MPU6050 in the current cycle, with a sampling period of 0.02 s.

## 4. Experiment

The size of the experimental pool is 2.5 m × 1.6 m, and the water depth is 0.6 m. The experimental parameter settings are shown in Table 2.

### 4.1. Yaw Control

In Section 3, the path-tracking problem is theoretically derived to be transformed into a yaw-tracking control problem. The internal control system is tasked with guiding the robotic fish to follow the real-time desired yaw-angle output by the outer guidance subsystem, thereby achieving effective path-following control. Consequently, it is imperative to conduct tests on the efficacy of the internal control system in order to validate the feasibility of the proposed control scheme. During the path-following process, the desired yaw angle provided by the guidance subsystem continuously changes, it becomes essential to assess and test both constant and dynamically changing yaw angles for their trackability by our proposed controller. As a result, the yaw-tracking control experiment is divided into two parts, namely tracking of static yaw angles and tracking of dynamic yaw angles.

**Figure 7:**
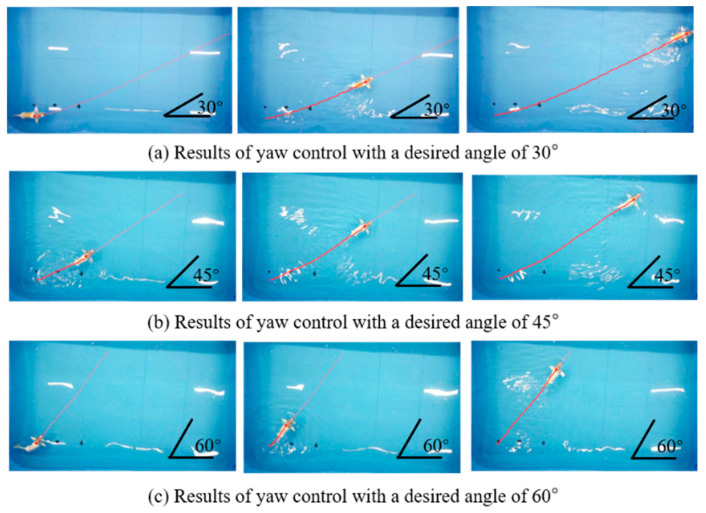
Results of directional tracking experiment.

**Figure 8:**
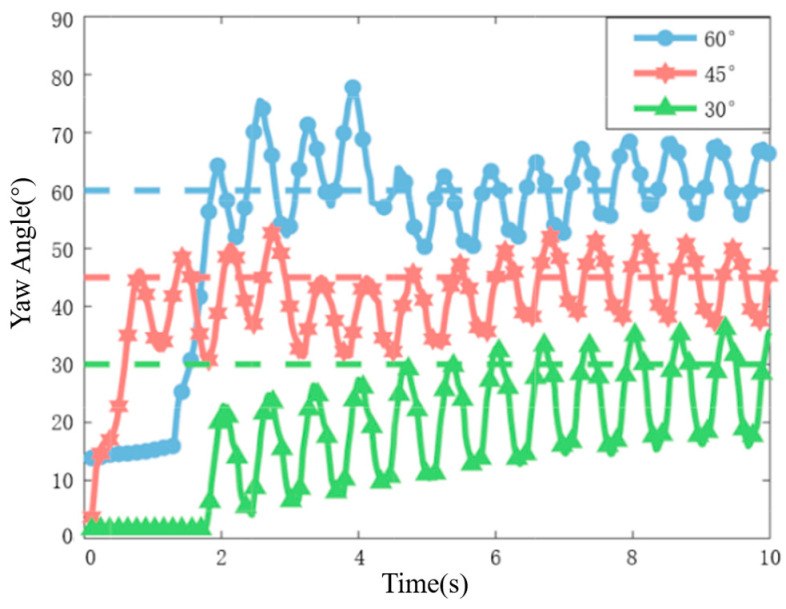
Variation of actual yaw angle with different desired angles.

In the static yaw-angle tracking experiment, we conducted tracking for angles of 30°, 45°, and 60°. The trajectory of the robotic fish during the experiment is illustrated in Figure 7. A careful examination of the angle diagram in the bottom right corner of the figure reveals a close alignment between the fish’s movement direction and the desired angles. To provide a more intuitive representation of the relationship between actual and desired values, Figure 8 displays the real-time yaw angle of the fish compared to the desired yaw angle. Remarkably, the implementation of the proposed incremental PID yaw tracking controller results in effective tracking performance for static yaw angles in robotic fish.

**Figure 9:**
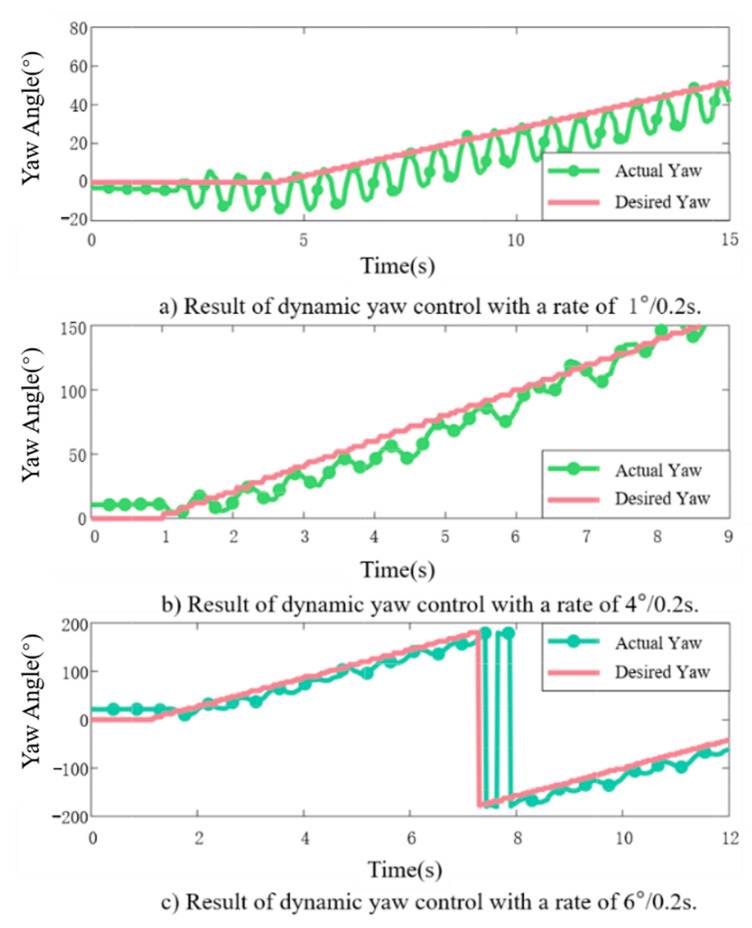
Variation of actual yaw angle for desired angles with different rates of change.

In the experiment of dynamic yaw-angle tracking, we designated the desired yaw angle as a linear time-varying variable, with incremental changes set at 1°/0.2 s, 4°/0.2 s, and 6°/0.2 s, respectively. The comparison between the actual yaw angle of the robotic fish and the time-varying desired yaw angle during the experiment is illustrated in Figure 9. It is evident that the proposed PID incremental controller demonstrates commendable tracking performance for dynamic yaw angles with varying angular growth rates.

Due to the inherent limitations of the fish’s bias, there is a slight delay in tracking the dynamic yaw angle with an angular growth rate of 6°/0.2 s, which represents the maximum limit that the fish can effectively track. However, during the path-tracking stage, the desired yaw-angle changes typically do not exceed 3°/0.2 s. Consequently, adopting the incremental PID controller as the inner-loop controller for path following presents a practical and viable solution.

### 4.2. Path-Following Control

The control block diagram for the path following is shown in Figure 6, where the real-time position of the robotic fish serves as feedback. However, traditional positioning modules are not suitable due to the physical dimensions of the fish and constraints imposed by the operating environment. Therefore, this study utilizes real-time experimental images captured using a global camera, with image-processing techniques employed to extract the fish’s position. Considering both the image-processing stage and the characteristics of the robotic fish, increasing the control frequency does not significantly improve performance. Due to limitations in the fish’s actuators, instantaneous mode transitions cannot be achieved. As a result, the control period is set to 200 ms in this study, taking into consideration the requirements of the image-processing stage and the fish’s actuation capabilities. Unlike trajectory tracking, path-following control is not constrained by time but rather focuses on ensuring that the robotic fish can move along a predetermined path. Therefore, to simplify the control implementation, in this study, the parameters M, ω, and R in the CPG high-level control commands (M,B,ω,R) are set as constants for each experiment. The fish only adjusts the bias of the caudal fin (B) during the tracking process to achieve turning. Specifically, the beating frequency (ω) is set to 1.5 Hz, the time ratio (R) is set to 1, and the oscillation amplitude (M) is set to 10.

The initial position of the robotic fish is set to (250,150), and the target path is set as $x_{P}\left(\varpi \right)=1000+300cos(2\pi \varpi )$, $y_{P}\left(\varpi \right)=500+300sin(2\pi \varpi )$ with $\varpi \left(0\right)=0$.

**Figure 10:**
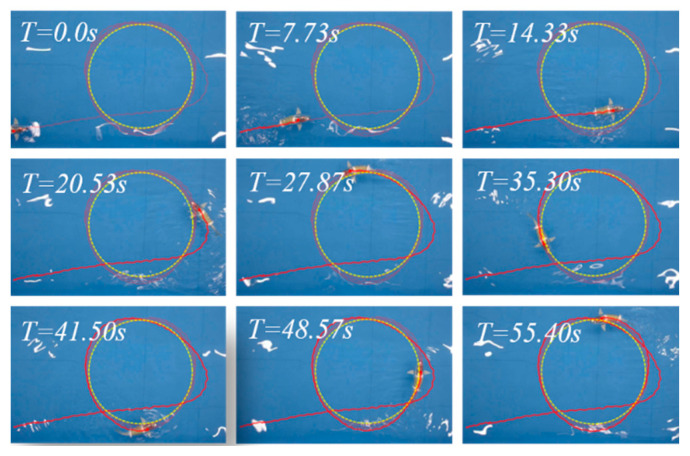
Snapshot sequence for circle path following. The yellow dashed line represents the expected trajectory, while the red implementation represents the actual trajectory of the robotic fish.

Figure 10 depicts a series of snapshots demonstrating the path-following process guided by the proposed strategy (Video S1). The anticipated path is denoted by a yellow dotted line, while the actual swimming trajectory of the robotic fish during the experimental trial is represented by a red solid line. It can be observed from Figure 10 that the robotic fish enters the tracking phase at T = 14.33 s. At this point, the controller initiates the turning motion because there is a significant difference between the heading angle of the robotic fish and the path angle. However, due to the inherent control lag, the robotic fish deviates from the desired path between 14.33 s and 20.53 s. Subsequently, under the guidance of our proposed strategy, at T = 20.53 s, the robotic fish successfully returns to the proximity of its intended path and maintains stability with minimal deviations from it thereafter.

**Figure 11:**
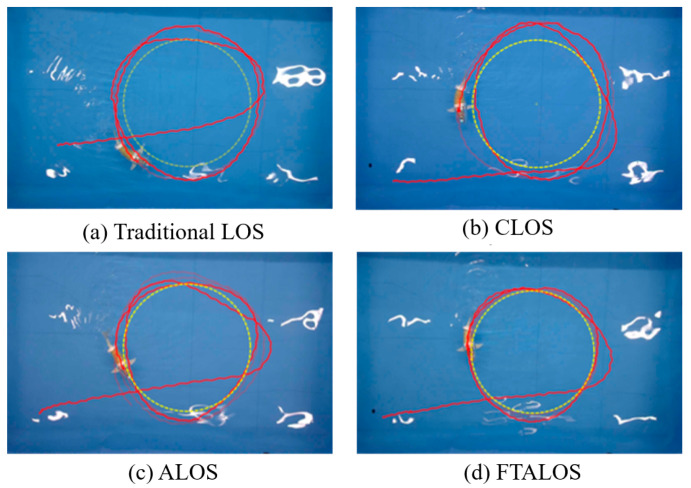
Comparison of the actual trajectories and desired paths under different guidance strategies. The yellow dashed line represents the expected trajectory, while the red implementation represents the actual trajectory of the robotic fish.

A comparison was made between the traditional fixed lookahead distance LOS, enclosed circular lookahead distance (CLOS), and the proposed ALOS in this study. Figure 11 shows the comparison of the path-following experiments using different strategies, depicting the actual swimming trajectories of the robotic fish under each navigation method. It can be seen that the traditional LOS is slow to converge and prone to oscillation in the process of path following, which is caused by the inherent limitations of the traditional LOS, i.e., drawback 3 of the traditional LOS, as mentioned in this paper. Too large a lookahead distance will result in slow convergence, while too small a lookahead distance will result in oscillation in the vicinity of the trajectory, making it difficult to obtain the appropriate lookahead distance. Similarly, CLOS has significant overshooting but performs a little better than LOS at sharp corners. In contrast, the ALOS proposed in this paper significantly outperforms the previous two in both sharp corners and the amount of overshooting and tracks the desired path more smoothly, which is due to the fact that the ALOS can adaptively adjust the lookahead distance. But, the path-tracking accuracy is still insufficient, due to the interference from the sideslip angle of the robotic fish. However, the FTALOS strategy proposed in this paper not only adaptively adjusts the lookahead distance but also takes into account the interference of the sideslip angle of the robotic fish, estimates the side-slip angle, and obtains a more realistic line-of-sight angle, and the final experimental results are satisfactory and significantly better than the first three.

**Figure 12:**
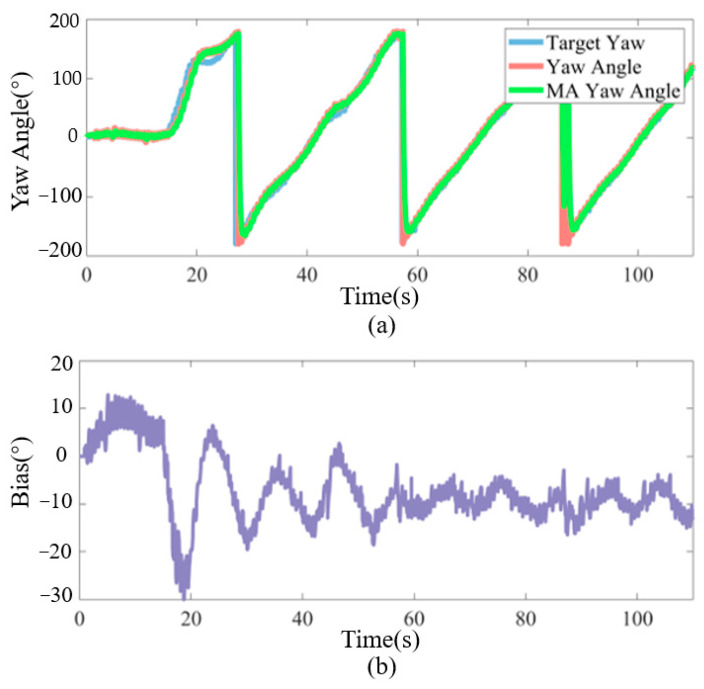
Experiment results. (**a**) Comparison of target yaw angle and actual yaw angle; (**b**) real-time bias of CPG high-level command, it represents the swinging center of the caudal fin of robotic fish.

Figure 12a depicts the real-time variation of the yaw angle during the experimental process, demonstrating that the FTALOS continuously updates the desired yaw angle. The robotic fish achieves accurate tracking of this signal under the influence of the incremental yaw-tracking PID controller, thereby accomplishing the path-tracking task. Figure 12b presents the tail-fin deflection signal in the CPG high-level control command throughout the experiment. When combined with Figure 10, it becomes apparent that, during the initial stage, slight turns are executed by the robotic fish to reach the starting tracking point, resulting in a small positive deflection value. However, at T = 14.33 s, a substantial turn is undergone by the robotic fish, leading to a rapid transition of the deflection value to a larger negative magnitude. At T = 20.53 s, the robotic fish successfully returns to the desired path and, thereafter, swims along the desired trajectory, gradually stabilizing the subsequent deflection value, which consistently hovers around −10.

**Figure 13:**
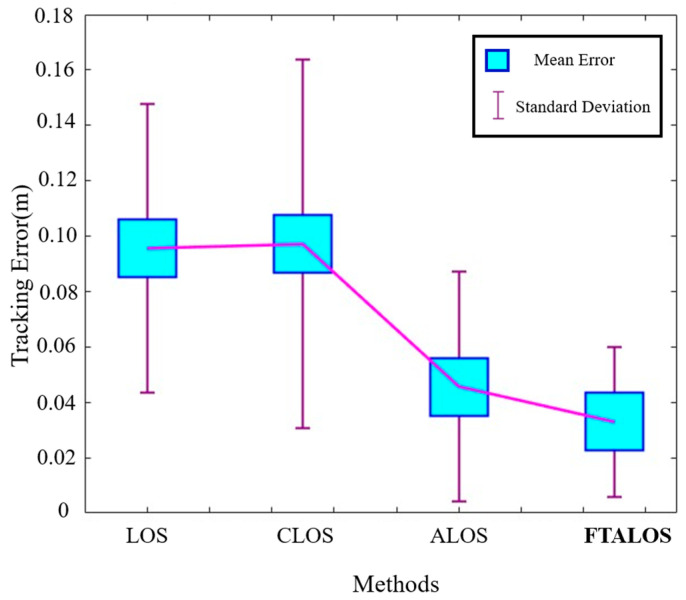
Tracking-error comparison of different control methods.

To quantitatively assess the advantages of the proposed FTALOS, we measured the experimental tracking errors, as presented in Table 3 and Figure 13. The results demonstrate that the FTALOS exhibits superior tracking performance across all three error dimensions, including RMSE, MSE, and MAE. Specifically, it achieves the lowest RMSE value of 0.0544, the lowest MSE value of 0.0425, and the lowest MAE value of 0.0030. Meanwhile, Figure 13 clearly shows that FTALOS has the smallest average tracking error and standard deviation. This indicates that the FTALOS outperforms both the CLOS and traditional LOS methods. The ALOS guidance strategy, incorporated in the FTALOS, enables faster convergence to the desired path compared to the traditional LOS, which exhibits slower convergence speed. Additionally, the inclusion of FTO enhances the performance of the ALOS, further improving its tracking capabilities.

However, our proposed FTALOS path-tracking method has certain limitations as it relies on the assumption that the path is expected to be second-order differentiable. Consequently, the tracking results may not meet expectations for paths with folded lines.

## 5. Conclusions

In this paper, an FTALOS guidance subsystem is proposed to achieve a planar path following a bio-inspired robotic fish equipped with a compliant and flexible tail fin driven by a cable mechanism. Initially, the kinematic model of the underwater bio-inspired robotic fish is introduced based on coordinate transformations. Subsequently, an ALOS guidance strategy is proposed, which incorporates compensation for the sideslip angle. Moreover, an incremental PID controller for yaw-tracking control is introduced and experimentally validated to evaluate its ability to track the yaw angle. The integration of the guidance subsystem and the PID heading controller is synergistically implemented, and the efficacy of the proposed tracking strategy is verified through experiments. The experimental results demonstrate that the robotic fish guided by the proposed FTALOS exhibits minimal overshoot and achieves a lower steady-state error compared to the traditional LOS and CLOS.

In the future, we will develop advanced mechanical structures to enhance the swimming efficiency and experimental reproducibility of robotic fish. Moreover, our focus will remain on refining control algorithms, including model-free control based on reinforcement learning, to enable robotic fish to autonomously learn and improve controller robustness. Furthermore, we will conduct outdoor experiments with random perturbations to track more complex paths.

## Figures and Tables

**Table 1:** The electronic components used in the robotic fish.

Items	Characteristics
Microcontroller	STM32F103
Servo motor	SAVOX SW-1210SG
Communication module	E62-433T20D
Inertial Measurement Unit (IMU)	MPU6050
Power Supply	DC 7.4 V

**Table 2:** Parameters setting for experiment.

$dh_{max}$	$dh_{min}$	k	r	$K_{P}$	$K_{I}$
150	30	60	20	0.6	0.0001
$K_{D}$	λ	M	B	ω	R
0.0003	1.5	10	-	1.5	1

**Table 3:** Quantitative analysis of circle path-following error for experiments.

Items	Quantitative Analysis(m)		
	RMSE.	MAE	MSE
Traditional LOS	0.0986	0.0880	0.0097
CLOS	0.0980	0.0831	0.0096
ALOS	0.0706	0.0550	0.0050
FTALOS	**0.0544**	**0.0425**	**0.0030**

## Data Availability

No new data were created or analyzed in this study. Data sharing is not applicable to this article.

## Supplementary Materials

The following supporting information can be downloaded at: https://www.mdpi.com/article/10.3390/biomimetics9090556/s1, Video S1: demo video.
